# Giraffe Stature and Neck Elongation: Vigilance as an Evolutionary Mechanism

**DOI:** 10.3390/biology5030035

**Published:** 2016-09-12

**Authors:** Edgar M. Williams

**Affiliations:** Faculty of Life Sciences and Education, University of South Wales, Wales CF37 1DL, UK; Mark.Willaims@Southwales.ac.uk; Tel.: +44-1443-483-893

**Keywords:** feeding giraffe, sexual selection, okapi, silvatherium, thermoregulation

## Abstract

Giraffe (*Giraffa camelopardalis*), with their long neck and legs, are unique amongst mammals. How these features evolved is a matter of conjecture. The two leading ideas are the high browse and the sexual-selection hypotheses. While both explain many of the characteristics and the behaviour of giraffe, neither is fully supported by the available evidence. The extended viewing horizon afforded by increased height and a need to maintain horizon vigilance, as a mechanism favouring the evolution of increased height is reviewed. In giraffe, vigilance of predators whilst feeding and drinking are important survival factors, as is the ability to interact with immediate herd members, young and male suitors. The evidence regarding giraffe vigilance behaviour is sparse and suggests that over-vigilance has a negative cost, serving as a distraction to feeding. In woodland savannah, increased height allows giraffe to see further, allowing each giraffe to increase the distance between its neighbours while browsing. Increased height allows the giraffe to see the early approach of predators, as well as bull males. It is postulated that the wider panorama afforded by an increase in height and longer neck has improved survival via allowing giraffe to browse safely over wider areas, decreasing competition within groups and with other herbivores.

## 1. Introduction

Giraffe (*Giraffa camelopardalis*), as African megaherbivores, are the tallest mammals, with both long necks and legs. Its neck is the longest on any extant animal at around 2–3 meters in the largest males [1]. Other animals with long necks relative to their body size are the camel, llama and several bird species like swans and ostrich. Longer necks were found on the now extinct sauropods whose necks reached up to 15 meters [2]. Apart from the long neck, the total height of the giraffe is further increased by its long legs, these greatly add to its height, allowing a mature bull to reach 5 meters in height [3]. This extraordinary body plan has required unique adaptions to three physiological systems, the cardiovascular, musculoskeletal and nervous system [4,5,6,7].

The giraffe’s early ancestors had short necks. The early giraffe fossil record is extensive and shows that its primary ancestors were distributed across Asia, Europe and Africa, and its recent ancestors looked more like the modern day moose or elk [8,9,10]. This ancestral heritage is manifest in the only extant relative of the giraffe, the okapi (*Okapia johnstoni*). This is a diminutive species in comparison, first described scientifically in 1901, and exclusively a forest bound animal. The okapi is a solitary animal and, in its natural environment, is surrounded by a plentiful supply of vegetation within easy reach, so a long neck provides no particular survival advantages [11]. Indeed, being tall and long-necked in a verdant forest environment may be a considerable hindrance and generally not improve survival [11]. The fossil record gives a clear route from the ancestors such as the Pleistocene giraffe, silvatherium to the okapi, all with relatively short necks. Some long-necked *Giraffa* appear in the fossil record around eight million years ago, such as the *Bohlinia attica* from the areas of Pikermi and Samos [10]. The fossil record is poor for the current giraffe species and there are no progressive examples of a fossil giraffe with necks of ever-increasing length [12]. However, recent evidence suggests that the elongation evolved first through cervical vertebral elongation, followed by caudal lengthening of the vertebrae [12,13]. Therefore, clues of the evolutionary drivers for neck elongation and height are not available in the fossil record and current knowledge of giraffe biology and behaviour has been used to postulate the drivers (Table 1) [1]. This review explores a number of proposed and valid evolutionary mechanisms for neck elongation and the increase in stature, and revisits the role that vigilance, and in particular horizon vigilance may have on this selection.

## 2. High Browse

The first serious attempts to explain the giraffe’s long neck were made by Jean–Baptiste Lamarck (1744–1829) in 1809, who postulated, that a long neck was an acquired characteristic. Elongation being gained by the giraffe constantly stretching its neck to eat the highest foliage available, a trait then passed on to its offspring [14]. Charles Darwin (1809–1882) provided a more plausible mechanism in his Origin of Species, by postulating that elongation occurred through the inheritance of traits; the giraffe most likely to survive and breed were those that could feed on the highest vegetation, especially when in short supply and unavailable to other short neck herbivores [15,16]. Here, competition for food led to neck elongation. Neither authors suggest why this solution was followed only by the giraffe. Darwin’s view is still the most popular today, as giraffe and elephants are the only animals that feed from tall trees on the arid savannah, with many of the tallest trees uniquely sculpted by browsing giraffe. However, this mechanism does not explain their extreme height, as giraffe at half their current height would still be able to out-reach its fellow folivores [17].

## 3. Sexual Selection and Differentiation

Male giraffe fight for dominance using their elongated muscular necks, large bony heads and ossicones. From an early age males spar with one another using their heads to take swinging blows at their opponent’s body. In young males it is a playful behaviour but in adult males this behaviour can lead to severe injuries and death. Adult males have up to five horny rounded protuberances, ossicones, on top of their heads, which can inflict more harm. Sparring with a head weighing up to forty kilograms, requires a strong neck, which can itself weigh over 100 kilograms [6]. It is posited that the tallest giraffe, with the longest necks will be the most successful to breed and therefore be most likely to pass their traits onto the next generation [18]. Females rarely partake in this behaviour, but when they do it is only with males, so it should not be selective in females. The evidence for this mechanism of sexual selection is weak as there are no great differences between male and female giraffe necks, when scaled for gender body size [17,19,20,21].

## 4. Thermoregulation

For giraffe, being tall and presenting a narrow shape to the sun provides the creature with a thermoregulatory advantage [22]. However, for this to be an evolutionary drive for neck elongation, the neck should be a good thermal regulator, be free of fur and have a skin with a good blood supply. This is not the case and so does not support this mechanism [1]. The giraffe’s unique skin pattern of dark and light patches is an aid to improving thermoregulation, but again this would not provide selection for increased neck elongation or overall stature, and would result in an increase in body surface area. In terms of thermoregulation, most large ungulates have increased in size laterally rather than in height to achieve this.

## 5. Climate Change

Giraffe are sensitive to their environment and live in arid regions, as reflected in their current distribution in continental Africa [23]. In the late Miocene, when the climate was wetter, there were many *Giraffid* species across Asia and Europe; most species had short necks and different dietary profiles, as not all were browsers like today’s two species [8,9]. A change in climate altered the vegetation at the end of the late Miocene (six million years ago), away from tropical vegetation to open-woodland savannah [24]. This change would have favoured browsers. How adaptation to this drier climate favoured the browsers with longer necks and larger mouths needs further investigation, as this would have been different in the current giraffe sub-species [25]. 

## 6. Vigilance and Horizon Scanning

In herbivorous ungulates, prey vigilance, especially while feeding, is important for survival and breeding. It can therefore be postulated that in giraffe an increase in height and the possession of a flexible long neck would be biologically advantageous by allowing the approach of potential predators or suitors to be spotted earlier [26]. Here, vigilance has two components, one is the area of the visual field (or extent of the immediate horizon) and the other is the action of vigilance itself, a specific behaviour which requires focus, cognition and time. Giraffe with their eyes at 3–5 meters above ground level will be able to see further than their fellow herbivores whose eyes are situated only 1–2 meters above ground level. Vigilance behaviour and social interaction during feeding in giraffe has been studied by Cameron and Du Toit [27], while their level of vigilance during drinking at a waterhole in the presence of lions was studied by Périquet et al. [28]. Giraffe vigilant behaviour, is defined by the animal standing still, scanning with its eyes, listening with its ears facing forward and raising its head [28].

When feeding in small or large groups, both male and female giraffe are equally vigilant, and it is seen as a distraction from feeding, thus entailing a negative cost [27]. Giraffe are more watchful of their neighbours rather than other species present, and especially so when a bull giraffe is present [27]. This suggests that vigilance is important in regulating social contact. Giraffe are polygynous and asynchronous breeders, and as such not all females in a group would be in oestrus [3]. Bull giraffe will seek-out receptive females by sampling their urine which can interfere with their feeding. Thus a female giraffe who is not in oestrus can maximise her feeding by moving away from any approaching bull giraffe. The negative cost of vigilance while browsing would be offset by avoiding the unwanted interruption of the bull giraffe.

While drinking, giraffes are at their most vulnerable to predation by lions because of their “splayed leg” stance. It has been suggested that because of its long legs a giraffe would not be able to drink without a long neck [2]. A long-legged short neck giraffe could most likely drink on its knees, so neck elongation is not likely a result of long legs alone. As expected and like other herbivores, giraffe vigilance while drinking increases in the presence of lions [28]. Even with splayed legs, the head can be raised, and a longer neck helps extend the visual horizon, allowing approaching lions to be spotted earlier. The extra visual horizon gained provides a survival advantage.

Bull giraffe lead a largely solitary existence, but still need to be vigilant of predators, other giraffe, such as rival bulls and females for mating. In the solitary bulls, a longer neck would favour the evolution of neck elongation as the tallest giraffe would be able to scan a more distant horizon and therefore be forewarned of any potential threat, gaining an advantage over a shorter rival.

While vigilance distance is enhanced by having eyes placed 3–5 meters above the ground, and provides a good platform for horizon scanning, it is a disadvantage for viewing close surroundings particularly the immediate area around the feet. This could be a considerable disadvantage when the giraffe is running, as failure to avoid objects on the ground could be disastrous if escaping a predator. The giraffe’s eye has adapted, giving it a dual purpose, and has the ability to see long distance while at the same time observing the ground immediately in front of itself [25,29].

The evolutionary development of long necks in two closely related species, camels and llamas cannot be explained by the high browse or sparring hypotheses. However, an increased visual horizon could be a positive driver for developing a longer neck, especially when living in open areas. In birds such as ostrich, neck elongation is extreme [30]. Ostrich spend timing sitting on their nests close to the ground, and need to watch for predators, again being able to spot a predator at a greater distance provides survival advantages. The sooner a predator is spotted the sitting bird can either conceal itself closer to the ground or leave the area [27,28,29,30,31]. 

In sauropods, dinosaurs with a very long neck, vigilance is unlikely to have been an important driver for neck elongation, it is more likely access to food, having a longer neck increases the feeding arc, the wider the arc the less often the animal has to relocate itself, providing an energy advantage. It is thought that these animals were unable to lift their heads much above the horizontal [32]. A horizontal stance will also negate the potential for a high arterial blood pressure as is seen in the giraffe.

The skeletal anatomy of the head and neck give the giraffe the ability to move their heads in a wide arc both vertically and horizontally. This has advantages when feeding on trees, as it imparts a greater reach, and allows the animal to maximise its foraging before moving to another site. The giraffe also has one of the longest tongues and largest mouth volumes of any mammal with the prehensile tongue allowing the giraffe to extend its reach both horizontally and vertically. This adaption can be seen as an extension of the high browser hypothesis, but feeding while standing in a “safe-spot” is another reinforcement of the idea that far-seeing drives giraffe behaviour and survival against predation.

Vigilance and reach is improved with increased height. Neck elongation is ultimately limited by the musculoskeletal support of the neck and head, and the need to overcome gravity as increasing the distance between the heart and brain requires an ever increasing arterial blood pressure and extensive modification of the vasculature control mechanisms to prevent blood pooling [5]. Another way of increasing overall height is to elongate the legs. This in itself has limitations in that bone weight would become inhibitive above a certain size. Although the cardio-cerebral distance is not increased when standing, it is when the giraffe lowers its neck to drink that longer legs would increase the cardio-cerebral distance. The extra blood pressure experienced by lowering its head is offset by a splayed leg stance; but the giraffe still needs to regulate upper body blood pressure during this manoeuvre. Thus increasing height via longer legs has disadvantages as well as advantages and it is a balance between these that will dictate their maximum height.

## 7. Conclusions

Recent morphological and genetic studies in giraffe and okapi have shown that adaptions to the muscles, bones, neural and cardiovascular systems are driven by unique mutations to the giraffe genes controlling growth and the cardiovascular system [4,33]. The ability to view an extended horizon offers giraffe the opportunity to maximise their foraging by firstly giving the animal a better understanding of their immediate surroundings in terms of sources of food supply, the distribution and proximity of fellow giraffes and potential predators. This extra height does not alter vigilant behaviour as this is independent of height as studies have shown. The okapi living in a dense tropical environment which does not benefit from “horizon-vigilance” has an unremarkable neck and height. Thus, the average height of a specific giraffe population should be inversely dependent on population density and the openness of savannah. In more open savannah and low population density, taller giraffe would be favoured if horizon vigilance were a significant driver of the evolution of neck elongation and stature.

## Figures and Tables

**Table 1:** Explanations for giraffe neck elongation and stature (modified from Wilkinsons and Ruxton 2011, [1]).

Mechanisms for Neck Elongation and Increased Height
High browse
Sexual selection
Thermoregulation
Climate change
Leg-length
Horizon vigilance
